# Does modulation of tau hyperphosphorylation represent a reasonable therapeutic strategy for Alzheimer’s disease? From preclinical studies to the clinical trials

**DOI:** 10.1038/s41380-023-02113-z

**Published:** 2023-06-02

**Authors:** Neha Basheer, Tomáš Smolek, Imtaiyaz Hassan, Fei Liu, Khalid Iqbal, Norbert Zilka, Petr Novak

**Affiliations:** 1 Institute of Neuroimmunology, Slovak Academy of Sciences, Bratislava, 845 10 Slovakia; 2 Centre for Interdisciplinary Research in Basic Sciences, Jamia Millia Islamia, Jamia Nagar, New Delhi 110025 India; 3 Department of Neurochemistry, Inge Grundke-Iqbal Research Floor, New York State Institute for Basic Research in Developmental Disabilities, 1050 Forest Hill Road, Staten Island, NY 10314 USA; 4 AXON Neuroscience R&D Services SE, Bratislava, 811 02 Slovakia; 5 AXON Neuroscience CRM Services SE, Bratislava, 811 02 Slovakia

**Keywords:** Drug discovery, Neuroscience

## Abstract

Protein kinases (PKs) have emerged as one of the most intensively investigated drug targets in current pharmacological research, with indications ranging from oncology to neurodegeneration. Tau protein hyperphosphorylation was the first pathological post-translational modification of tau protein described in Alzheimer’s disease (AD), highlighting the role of PKs in neurodegeneration. The therapeutic potential of protein kinase inhibitors (PKIs)) and protein phosphatase 2 A (PP2A) activators in AD has recently been explored in several preclinical and clinical studies with variable outcomes. Where a number of preclinical studies demonstrate a visible reduction in the levels of phospho-tau in transgenic tauopathy models, no reduction in neurofibrillary lesions is observed. Amongst the few PKIs and PP2A activators that progressed to clinical trials, most failed on the efficacy front, with only a few still unconfirmed and potential positive trends. This suggests that robust preclinical and clinical data is needed to unequivocally evaluate their efficacy. To this end, we take a systematic look at the results of preclinical and clinical studies of PKIs and PP2A activators, and the evidence they provide regarding the utility of this approach to evaluate the potential of targeting tau hyperphosphorylation as a disease modifying therapy.

## Introduction

Neurofibrillary pathology constitutes one of the two major histopathological hallmarks of Alzheimer’s disease (AD). The pathology is primarily composed of truncated and aberrantly hyperphosphorylated protein tau in the form of paired helical filaments (PHFs) or straight filaments (SFs) [1–7]. Remarkably, the density and stereotyped spatiotemporal distribution of this neurofibrillary pathology consistently correlate with the degree of cognitive decline, memory impairment, and brain atrophy [8–13]. Tau positron emission tomography (Tau-PET), and cerebrospinal fluid (CSF), and plasma biomarkers of tau further complement these findings [14–18]. On that account, tau pathology and subsequent neurofibrillary degeneration appear to play a leading role in the pathophysiology of AD.

Tau, recognized as an intrinsically disordered protein (IDP), undergoes various order-to-disorder or disorder-to-order transitions while retaining a flexible conformation. This flexibility is essential for its role in various cellular processes, such as regulation of microtubule (MT) dynamics, MT-mediated axonal transport, mRNA translation, cellular signalling, chromatin remodelling, neuroprotection, and neuronal development [19–28]. The monomeric conformational tau ensemble is modulated by a variety of factors, such as degradation processes, chaperone-mediated refolding, and several post-translational modifications (PTMs) [29–32]. In physiology, these modulations aid dynamic stability; in pathology, however, genetic mutations or dysregulation in these modulation results in weaker interactions between tau and its natural binding-partners, resulting in its accumulation. This creates conditions favourable for its unfolding, refolding, and misfolding into a tremendously large conformational ensemble that could potentially be capable of template-directed misfolding and aggregation [33, 34].

**Fig. 1:**
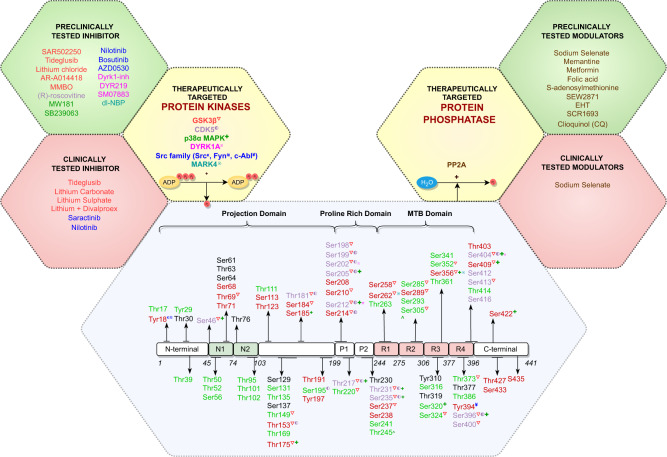
Schematic showing 2N4R tau (441 amino acids), the longest isoform expressed encompassing major structural domains. The phosphosites identified for AD pathology (in red), physiology (in green) and overlapping sites for both physiology and pathology (in purple) are shown here. Therapeutically targeted protein kinases (PKs) and protein phosphatase (PPs) are colour coded to their respective inhibitors or modulators explored clinically or preclinically.

Phosphorylation of tau is one of the most actively investigated PTMs, with significant impact on solubility, localization, function, interaction with other proteins and susceptibility to additional PTMs [35, 36]. The longest of the ‘classic’ six human tau isoforms (tau40, 2N4R) encompasses ~85 potential serine (Ser), threonine (Thr), and tyrosine (Tyr) phosphosites [37], mainly localized in the proline-rich region (residues 172–251) and the C-terminal tail region (residues 368–441) [37]. Relatively few phosphosites, but important in the context of pathology, are also present in the microtubule-binding region (MTBR; residues 244–369) (Fig. 1) [38–40]. In healthy individuals, only two to three phosphate molecules were detected per molecule of tau; in AD, this stoichiometry is increased manifold. Further data also suggest phosphorylation to be sufficient for the induction of tau filament formation [41–43]. The characterization of tau filaments via electron cryo-microscopy (cryo-EM) has revealed the presence of unique conformational folds that are conserved among individuals with the same tauopathy [44, 45]. These conformational folds are reported to encompass unique site-specific phosphorylation signatures [46–48], further suggesting that there may be a causative link between dysregulation of tau phosphorylation/dephosphorylation and different tauopathies.

A well-grounded rationale for support modulating tau phosphorylation by targeting protein kinases (PK) or protein phosphatase 2A (PP2A) to counteract AD. Although preclinical efficacy studies provide promising data, most if not all the clinical trials testing PKIs in AD failed to demonstrate any efficacy in humans, with only a few still unconfirmed positive trends. Other tau-targeted therapies in AD include active/passive immunization to target pathological tau species, and a range of small molecules intended to either reduce tau expression, aggregation, and propagation, or promote MT stabilization and tau degradation [49–51]; whether they are efficacious has not been conclusively assessed yet.Therefore, the aim of this study is to identify whether inhibition of tau hyperphosphorylation is a promising strategy for the development of disease-modifying therapies (DMTs) against AD.

## Protein kinases selected as a target for Alzheimer’s therapy

In AD, a concerted activity of several PKs is known to phosphorylate tau at nearly 40 AD-relevant epitopes [38, 39] (Table 1). PKs are stratified based on their proline-, non-proline -, or tyrosine-directed kinase activities.

**Table 1:** Protein kinases and their AD-relevant phosphosites.

Kinases	AD-relevant phosphosites
*GSK3*	Ser46, Thr69, Thr149, Thr153, Thr175, Thr181, Ser184, Ser198, Ser199, Ser202, Thr205, Ser210, Thr212, Ser214, Thr217, Thr220, Thr231, Ser235, Ser237, Ser258, Ser262, Ser285, Ser289, Ser305, Ser324, Ser352, Ser356, Thr373, Ser396, Ser400, Ser404, Ser409, and Ser413
*Cdk5*	Thr153, Thr181, Ser195, Ser199, Ser202, Thr205, Thr212, Ser214, Thr217, Thr231, Ser235, Ser396, and Ser404
*p38 MAPK*	Ser46, Thr69, Thr175, Thr181, Ser185, Ser202, Thr205, Thr212, Thr217, Thr231, Ser235, Thr245, Ser305, Ser320, Ser356, Ser396, Ser404, Ser409, and Ser422
*DYRK1A*	Ser202, Thr212, and Ser404
*MARK4*	Ser262 and Ser356
*Src*	Tyr18
*Fyn*	Tyr18
*c-Abl*	Tyr394
*ROCK*	Thr245, Thr377, Ser409

Proline-Directed Protein Kinases (PDPKs) target phosphorylation at serine and threonine that precedes a proline residue (Ser/Thr-Pro motif). Glycogen synthase kinase-3 (GSK3), cyclin-dependent protein kinase-5 (CDK5), p38 mitogen-activated protein kinases (p38 MAPK) and Dual specificity tyrosine-phosphorylation regulated kinase 1A (DYRK1A) belong to this PDPK subclass.

GSK3, with over 100 substrates, is a ubiquitously expressed multifunctional enzyme [52]. It regulates a wide array of molecular pathways, including glycogen synthesis, WNT signalling, transcriptional network maintenance, apoptosis, and axonal MT remodelling [53–59]. Expressed as two isoforms, α and β [60], it demonstrates an unusual preference for target substrates that are pre-phosphorylated or “primed” at Ser/Thr-XXX-Ser/Thr motifs. Tau fulfils this requirement, having 24 such motifs suitable for priming, facilitated by the activity of PKA, CK1, CK2, MAPKs, or CDK5 [61–63]. Differing in kinetics and epitope preferences, both isoforms phosphorylate tau in vitro [64–68].

Cdk5 has been demonstrated to be one of the most functionally diverse kinases within neurons. Cdk5 is crucial for a number of cellular and developmental processes, including neuronal migration, synaptic plasticity, microtubule regulation, pain signalling, and apoptotic cell death in neuronal diseases [69–73]. The expression is abundant in all tissue types, but its highest expression with associated kinase activities are primarily detected in the CNS [74]. Cdk5 complexing with neuron-specific regulatory subunits p35 and p39 plays a role in physiological tau phosphorylation. Under pathological conditions, cellular stress induces calcium-dependent protease calpain cleavage of p35 and p39 into p25 and p29, and a p10 fragment respectively. This results in pathological tau hyperphosphorylation [75, 76]. Cdk5 is involved in priming for GSK3β; their crosstalk is reported to be dependent on ageing [77]. Together with GSK3β, CDK5 activity is central to AD pathophysiology [77].

P38 MAPKs, regulate various cellular functions such as metabolism, secretion, migration, differentiation, apoptosis, and senescence in response to various extracellular stimuli [78]. In mammals, p38 MAPK is expressed as four isoforms: α, β, γ and δ [79], which differ in their expression patterns in neurons, astrocytes, microglia, and endothelial cells. However, they serve non-redundant functions (based on substrate specificities and sensitivities) depending on cell type and context [80]. P38 plays a physiological role in tau phosphorylation; however, in AD pronounced alteration in its levels and distribution is observed. It is capable of phosphorylating tau protein in vitro in a manner similar to the hyperphosphorylation of PHF-tau [81]. The finding that PHF-tau co-immunoprecipitates with p38, and that p38 co-purifies with PHF-tau, strongly suggests that they are physically associated [82].

DYRK1A is a dual-specificity proline/arginine-directed kinase that possesses both Tyr and Ser/Thr kinase activities. It plays a key role in neurogenesis, neuronal trafficking, aging, and outgrowth of axons and dendrites. *DYRK1A* is expressed ubiquitously, with the highest expression being observed in the brain and heart [83]. DYRK1A regulates numerous cellular pathways, among them tau phosphorylation [84, 85].

Non-Proline-Directed Protein Kinases (Non-PDPK) group comprises kinases that phosphorylate tau at Ser/Thr-X motifs. MAP/microtubule affinity-regulating kinase 4 (MARK4) belongs to this subclass.

MARK4, belongs to AMP-activated protein kinases (AMPKs) subfamily of calcium/calmodulin dependent protein kinases (CAMK). It plays a central role in regulation of cell shape and polarity during differentiation, chromosome partition in mitosis, and intracellular transport by acting on microtubule-associated proteins (MAPs), including MAP2, MAP4 and tau [86, 87]. It is predominantly expressed in the brain. MARK4-mediated phosphorylation subsequently catalyses the detachment of tau from MTs to regulate the transition between stable and dynamic MTs [88, 89]. De novo mutation in MARK4 is associated with early onset of AD, indicating the role of MARK4 in the development of AD [90]. Synergistic activity of Cdk5 with MARK4 is reported to augment tau phosphorylation at Cdk5 specific sites, in addition to those for MARK4, suggesting that Cdk5 could directly or indirectly phosphorylate and activate MARK4, creating a feedback loop [91, 92].

Tyrosine protein kinase (TPK) phosphorylates tau at Tyr18, Tyr29, Tyr197, Tyr310 and Tyr394. Src family kinase (SFK) members such as Src, c-Abl, and Fyn belong to this subclass of PKs.

Src is a proto-oncoprotein [93] that is ubiquitously expressed with low tissue specificity. Src is largely localized to the plasma membrane and cell junctions, with minor presence in nucleoplasm and cytoplasm where it interacts with different classes of cell receptors involved in signalling pathways that control immune response, cell adhesion, cell cycle progression, apoptosis, migration, and transformation [94]. Src interacts with the proline rich sequence in the N-terminus of tau [95] where it can bind and phosphorylate *Tyr18* [96, 97].

c-Abl is a proto-oncoprotein [98], alternative splicing of which results in two transcript variants, c-Abl-1a and c-Abl-1b [99, 100]. Its subcellular localization determines its function; cytoplasmic localization appears necessary for the transforming and cell survival functions whereas nuclear localization typically occurs in response to stress or overexpression and results in growth inhibitory functions, including cell cycle arrest and apoptosis [101, 102]. c-Abl is shown to co-immunoprecipitate with and directly phosphorylate tau [103] and modulate tau through activating Cdk5 by phosphorylating at Tyr15, as seen in AD models [104, 105].

Fyn is a non‐receptor or cytoplasmic TPK that mediates multiple transduction pathways in the central nervous system (CNS) including synaptic transmission, myelination, axon guidance, and oligodendrocyte formation. It is ubiquitously expressed with its highest expression seen in the CNS and lymphoid tissue. Levels of Fyn are reported to alter in AD [106]. Besides its commonly reported indirection associated with tau hyperphosphorylation, Fyn is shown to physically interact with tau and phosphorylate it at Tyr18 [107].

Rho-associated kinase (ROCK) belongs to the AGC (PKA/PKG/PKC) family of Ser-Thr specific PKs. It is regulated by the small GTPase RhoA and integrates activating signals from surface receptors, leading to both cell proliferation and migration [108]. RhoA/ROCK signalling pathway activation appears to affect a range of processes involved in the pathogenesis of AD, including tau hyperphosphorylation, Aβ aggregation, neuroinflammation, and synaptic damage, promoting neurodegeneration [109–112]. ROCK phosphorylates tau at *Thr245, Thr377, Ser409* and to some extent *Ser262* [113].

## Protein kinase inhibitors evaluated in AD

A large majority of PKIs investigated in AD with the intent of disease modification are designed/repurposed to target PKs to impede their enzymatic activity and thus reduce tau hyperphosphorylation. Here, we focus the discussion on those drugs that underwent clinical trials and briefly mention the ones in preclinical studies with the prime intention of targeting tau hyperphosphorylation.

*Saracatinib* (AZD0530) is a member of a large class of biologically active compounds called quinazolines, where quinazoline is substituted by (5-chloro-2H-1,3-benzodioxol-4-yl)amino, (oxan-4-yl)oxy and 2-(4-methylpiperazin-1-yl)ethoxy groups at positions 4, 5 and 7, respectively. It is a highly potent, orally available, small molecule that selectively inhibits the Src (Src, Fyn, Yes, and Lyn) and Abl families of tyrosine kinases [114, 115]. The compound was originally developed by Astra Zeneca as a treatment for various oncological disorders. Despite showing promising results in preclinical studies and a favourable safety profile, it was withdrawn in Phase 2 due to lack of efficacy. Clinical exploration of saracatinib in AD up to Phase 2a was conducted with the rationale to target Fyn kinase mediated Aβ toxicity. A separate line of evidence linked Fyn to tau; suggesting that the phosphorylation of tau at Tyr-18 was associated with the interaction of Fyn [107].

*Lithium (Li*^*+*^*)* salts are used for treating and preventing psychiatric disorders, primarily relapses in both type I and type II bipolar disorders (BDs), suicidal behaviours during major depressive disorders, and schizophrenic disorders. Li^+^ inhibits Mg^2+^-dependent enzymes by displacing Mg^2+^ from their binding sites within specific catalytic protein domains [116], thereby reducing the stability and activity of enzymes including GSK3 relevant both for neuropsychiatric and neurodegenerative disorders [117, 118]. Thus Li^+^ salts, such as lithium sulphate, lithium carbonate, and lithium chloride sparked substantial attention as a treatment possibility in AD. Chronic lithium treatment was shown to affect numerous tau kinases, with different biological effects depending on the concentration range and regional specificity [119]. However, the putative mechanism of action of lithium remains unknown.

*Nilotinib (AMN107),* an aminopyrimidine-derivative, is FDA approved drug for the treatment of Philadelphia chromosome positive chronic myeloid leukemia (CML). It is an orally available, selective, ATP competitive, reversible inhibitor of BCR/Abl tyrosine kinase with antineoplastic activity [120]. It was initially developed by Novartis Pharmaceuticals against imatinib-resistant mutants of BCR/Abl protein [121]. Low doses of nilotinib were further shown to penetrate the BBB, and reduce CSF tau, independent of Abl inhibition [122, 123]. The rationale to proceed for clinical use of nilotinib against AD was apparently based on the neuroprotective effects [124] and reduction of inflammation in non-tauopathic cellular and animal models [125, 126]. However, the availability of preclinical data that would show nilotinib counteracting tau hyperphosphorylation is limited, with such studies either not done or not reported.

*Tideglusib (NP031112/NP12)* belongs to the thiadiazolidine class of compounds; it’s composed of 1,2,4-thiadiazolidine-3,5-dione with a naphthalen-1-yl group at position 2 and a benzyl group at position 4. It is an orally active, potent, selective, irreversible, and non-ATP competitive small-molecule inhibitor of GSK3β developed against AD by the Zeltia group [127]. It’s considered a tau-protein kinase inhibitor with neuroprotective and anti-inflammatory effects [128, 129]. To the best of our knowledge there is no available in vitro data and merely a single in vivo study [130] showed clear reduction in tau hyperphosphorylation; several studies provide evidence of neuroprotection, and mitigating inflammation though [131].

*PKIs tested in preclinical studies.* Several potentially interesting compounds were tested for efficacy in both in vitro and in vivo studies. The compounds including SAR502250 [132], AR-A014418 [133, 134], MMBO [135], (R)-rescovitine [136], MW181 [137], SB239063 [137], bosutinib [138], SM07883 [139], Dyrk1-inh [140], DYR219 [141], BAY61-3606 [142] and Fasudil [143] are yet to be tested at clinical front for AD, whereas, lithium, tideglusib, nilotinib, and dl-3-n-butylphthalide have already made a headway to clinical trials.

## Involvement of protein phosphatase 2A (PP2A) in tau hyperphosphorylation

Protein Ser/Thr phosphatases comprise three major families: phosphoprotein phosphatases (PPPs), metal-dependent protein phosphatases (PPMs), and the aspartate-based phosphatases. Protein phosphatase (PP)1, PP2A, PP2B, and PP5 are PPPs that were found to dephosphorylate tau in vitro at various phosphorylation sites [144]. Among them, PP2A was shown to be the major phosphatase in the brain [145, 146] and accounts for >70% of total tau phosphatase activity [145]. Activity and/or expression of PP1, PP2A, and PP5 are decreased [145, 147–150], whereas PP2B truncation and activity are increased in AD brains [151].

PP2A regulates numerous signalling pathways, playing an important role in development, cell proliferation and death, cell mobility, cytoskeleton dynamics, and the control of the cell cycle [152]. PP2A is one of the most abundant enzymes, accounting for up to 1% of total cellular protein mass [153]. Its function is regulated by several endogenous proteins. PP2A dephosphorylates tau at almost all phosphorylation sites [145]. Among them, pThr205, pThr212, pSer262, and pSer409 are most conductive to PP2A activity [145]. The best characterized inhibitors of PP2A are PP2A-inhibitor 1 (I_1_^PP2A^, also known as Acidic Nuclear Phosphoprotein 32 A), PP2A-inhibitor 2 (I_2_^PP2A^, also known as SET), and CIP2A (cancerous inhibitor of PP2A).

PP2A activity is significantly decreased in the AD cortex and hippocampus [145, 147, 150]. The level of tau hyperphosphorylation at many sites is negatively correlated with PP2A activity [151]. Up-regulation of I_1_^PP2A^ and I_2_^PP2A^, and mislocalization and cleavage of I_2_^PP2A^ could underlie the inactivation of PP2A in neocortical neurons in AD [154]. Increased expression of CIP2A in the AD brain leads to tau mislocalization to dendrites and spines, and to synaptic degeneration [155]. Collectively, these studies directly point to a central role of PP2A dysfunction in AD pathogenesis, identifying PP2A as a druggable target of therapeutic interest [154, 156, 157]. Due to the pivotal cellular role of PP2A and its broad range of substrates, indiscriminately activating PP2A with potent compounds risks toxicity, though.

A comprehensive literature search was performed to identify pertinent in vivo efficacy studies utilizing PP2A activators such as antineoplastic cancer drug sodium selenate [158–161]; the anti-diabetic drug, glimepiride [162] and metformin [163–165]; a zinc ionophore and an experimental drug candidate, copper/zinc chaperone (PBT2) [166]; anti-depressant, S-adenosylmethionine [167–169]; anti-microbial clioquinol (an intracellular zinc chelator) [170] an immunomodulating medication, fingolimod [171]; and caffeine [172], demonstrated substantial reduction of tau phosphorylation, insoluble tau, and tangle load, along with the alleviation of cognitive/behavioural phenotypes (Supplementary Table S1). Even though the pharmacological modulation of tau pathology is evident from these efficacy studies, the precise mechanism of action (MOA) of most of these multi-targeted drugs remains elusive. For instance, the PP2A stimulator metformin can target an upstream regulatory kinase, AMPK, that can inhibit GSK3β, and thus the observed effects cannot be evenly segregated [173, 174]. Among the aforementioned PP2A activators, only sodium selenate stated PP2A activation as its MOA in clinical trials.

## Efficacy studies on selected kinase inhibitors

To evaluate in vivo efficacy of putative PKIs a wide range of preclinical studies has utilized aged, amyloid-beta (Aβ), Down syndrome, TMT chloride, colchicine, scopolamine, streptozotocin, anaesthesia, hypothermia, or hypoxia/stroke rodent models of AD [173–178]. Whereas on one hand, some of these models develop hyperphosphorylation, amyloid plaques (some transgenic models), and inflammation-induced synaptic dysfunction leading to visible behavioural deficits, they lack the targeted pathological hallmarks, i.e., NFTs. As a result, their relevance and translational value remain unknown.

We performed a comprehensive literature search to identify studies investigating the effects of PKIs exclusively in rodent tauopathy models (Tables 2). We collected pertinent publications from three independent databases: PubMed, Web of Science, and Google Scholar. We used “Alzheimer’s disease” AND “protein kinase inhibitors” as key words for the literature search. The search was restricted to English language and not by date. The inclusion criteria included: transgenic rodent models expressing mutated, truncated, or wild-type tau; original experimental studies; studies demonstrating target engagement, i.e., behavioural and biochemical/histological studies to demonstrate reduction in tau hyperphosphorylation. Exclusion criteria comprised duplicated references; review articles; literature with incorrect or incomplete data; absence of behavioural and biochemical/histological evaluation of tau phosphorylation.

**Table 2:** Preclinical studies to date exploring the effects of protein kinase inhibition on the level of pathological tau phosphorylation in tauopathy models^a^.

Kinase	Inhibitor/s	Age; sex; animal model	Administration; Dosage	Treatment^b^	Results			Ref.
					Behavioural test	Biochemical analysis	Histopathology/ Immunochemistry	
GSK3	SAR502250	3 m; F; JNPL3(P301L) mice	Orally; 1, 3, 10, 30 and 100 mg/kg	Once	NA	Dose dependent ↓ in pS396-Tau from 10 mg/kg/d onwards	NA	[132]
		6 m; M; APPsw-Tauvlw mice	Orally; 10 & 30 ml/kg	7w	Rescued impaired memory performance in NORT on both doses	NA	NA	
	NP12/ Tideglusib	9 m and 12 m; M,F; APPsw-tauvlw mice	Oral gavage; 200 mg/kg	12w	Improved escape latency in MWM but no change in swimming speed at 15 m	No change in levels of human-tau in soluble-tau preparation; ↓ pS202- & pS396/404-Tau in 15 m old mice by more than 60% whereas no change in 12 m old mice; As expected no change at pS422-tau; Aggregated filaments too low to be quantified in Sarkosyl extractions in both age groups	↓ pS202- & pS396/404 tau; ↓ astrocytes (GFAP), 33% at 12 m in EC, 40 and 31% at 15 m in the CA1 and the EC region; ↑ in the number of neurons in the EC and the CA1	[221]
	Lithium (LiCl)	2 m; M, F; PrP T44 mice	Orally; 2.0 g/kg chow	4 m	Improved clasping phenotype in tail-suspension test	↓ accumulation of insoluble Tau protein in the CNS at 6 m and 9 m; No alteration in Tau phosphorylation	Tau-positive spheroids ↑ until 6 m, ↓ dramatically by 9 m; Vacuolar lesions present at 9 m not at 6 m, reflect degradation of Tau-positive spheroids; No change in number of neurons in spinal cord	[180]
	Lithium (LiCl)	15 m; M, F; Homozygous 3xTg-AD mice	Intraperitoneally; 300 μl of 0.6 mol/l	4w	No improvement of working memory deficit seen in T-maze	No alteration in t-Tau levels; ↓ in pT181-(AT270), pS202/T205- pT231/S235- & pT212-, pS214-Tau	No alteration in somatodendritic Tau levels; ↓ in pT181-, & pS202/T205-Tau	[179]
	Lithium (LiCl))	8 m and 11 m; M,F; JNPL3(P301L) mice	Intraperitoneally; 10 μl of 0.6 mol/l	30d	NA	No alteration in level of t-Tau; ↓ pS202-, pS396/404-Tau; ↓ aggregated Tau in the Sarkosyl extract of cortex & brainstem, similar in M & F	No difference in grey matter pathology detected by Gallyas silver stain & MC1 immunostaining; ↓ levels of insoluble Tau stained with LFB; Positive correlation between insoluble Tau levels with MC1 staining and axonal degeneration	[133]
	AR-A014418	8 m and 11 m; M,F; JNPL3(P301L) mice	Oral gavage; 30μmol/kg	30d	NA	↓ pS396/404-Tau in brainstem	NA	
	MMBO	11 m and 13 m; M; 3xTg-AD mice	Orally; 3 mg/kg	Twice a day, for 33d and 22d	No improvement in Y-maze; ↑ frequency of exploration in NORT	↓ pT181-, pS199-, pT205-, pS202/T205-, pT231- Tau, and pS396-Tau in dose dependent manner; No inhibition of pS214-Tau observed	No change in total human; ↓ pS202/T205-Tau in a dose-dependent manner	[135]
CDK5	(R)-roscovitine	4 m; M,F; 3xTg-AD mice	Intraventricular infusion; 0.5 mg/kg	14d	NA	No alteration in level of t-Tau; ↓ 2x pS202/T205- and 3x pT231-Tau; No change in pS396/404-Tau levels	NA	[136]
p38MAPK	MW181 (or MW01-10-181SRM)	20 m; M,F; hTau mice	Oral Gavage; 1 mg/kg	14d	Improved spatial memory in the Y-maze test	↓ in levels of total in the Sarkosyl-insoluble fraction; ↓ in pT231- & pS202/T205-Tau	↓ in pS202/T205-Tau in the CA3/dentate gyrus; ↓ in pT231- Tau neurons	[137]
	SB239063	6 m & 20 m; M,F; hTau mice	Oral gavage; 5 mg/kg	14d	Improved spatial memory in the Y-maze test.	↓ in levels of Sarkosyl-insoluble Tau; No change in pS202/T205-Tau	↓ in pS202/T205-Tau in the CA3/dentate gyrus; No change in pT231-Tau neurons	
c-Abl	Nilotinib	1-6 m; M,F; Hemizygous and homozygous P301L mice	Intraperitoneal; 10 mg/kg	3w	NA	No change in t-Tau; ↓ pT181- & pS396-Tau; ↓ in levels of human Tau +H14:H15& pT231-Tau; ↑ autophagic flux i.e., ↓ in pre-lysosomal vacuoles AV10 & AV20 & ↑ lysosomal pT181-Tau	↓ in pS202/T205-Tau; ↓ pT231-Tau levels in cortex & hippocampus; ↓ degenerating fibers & neurons in cupric silver staining	[138]
cAbl/Src	Bosutinib	1-6 m; M,F; Hemizygous and homozygous JNPL3(P301L) mice	Intraperitoneal; 5 mg/kg	3w	NA	No change in t-Tau; ↓ pT181- & pS396-Tau; ↓ in levels of human Tau & pT231-Tau; ↑ autophagic flux i.e., ↓ in pre-lysosomal vacuoles AV10 & AV20 & ↑ lysosomal pT181-Tau	↓ in pS202/T205-Tau; ↓ pT231-Tau levels in cortex & hippocampus; ↓ degenerating fibers & neurons in cupric silver staining	
Fyn	AZD0530	11 m; NA; 3xTg-AD mice	Oral gavage; 5 mg/kg	Twice for 5w	NA	No change in levels of t-Tau, p-Tau (S199/S202 and S396) in TBS-soluble fractions; ↓ levels of t-Tau, p-Tau (S199/S202 and S396) in RIPA-extract of TBS-insoluble material	NA	[181]
DYRK1A	Dyrk1-inh	10 m; F; 3xTg-AD mice	Intraperitoneal; 12.5 mg/kg	8 w	No change in OFT. Improvement in RAWM	No alteration in level of t-Tau; ↓ levels of t-p-S396-Tau	NA	[140]
	DYR219	6 m; F; 3xTg-AD mice	Intraperitoneal; 12.5 mg/kg	3 m & 6 m	NA	↓ levels t-tau; ↓ levels of t-p-S396-Tau	No NFTs after 3 m and ↓ NFTs after 6 m	[141]
	SM07883	9 m; M,F; Tg JNPL3(P301L) mice	Oral; 3 mg/kg, b.w.	3 m; Every other day for 3 m	Improvement in motor functions and deficits in the wire-hang test	↓ in pT181-, pS202/T205-, pT212/S214-, pT231-, pS396-Tau; ↓ in sarkosyl-insoluble Tau fragments ↓ in GFAP expression by ELISA	↓ in astrocytes and microglia; ↓ percent in pS202/T205-Tau staining	[139]
MARK4	Dl-3-n-Butylphthalide (dl-NBP)	4 m; M,F; PS19 (P301S) mice	Oral Gavage; 30 mg/kg, b.w.	3x per 1w for 4 m	Partial improvement in escape latency in MWM; No change OFT; Increased freezing time in FCT	↓ pS262-Tau; No change in pS396-, pS404-, pT205-, pS199-, and pT231-Tau	↓ pS262-Tau; ↓ neuronal death; ↓ astrocytes and microglia	[182]
SKY	BAY61-3606 (BAY61)	30w; M,F; PS19 (P301S) mice	Intraperitoneal; 20 mg/kg	5 consecutive days/week for 3 m	Improvement in locomotor coordination, measured by a decreased latency to fall in the rotarod apparatus	↓ levels of t-Tau, p-Tau (S396/404 and S202), Tau oligomers, pS231-Tau and tau conformers in the detergent-insoluble fraction of brain homogenates; ↓ in pS231-Tau in detergent-soluble tau; ↓ neuronal loss; ↑ post-synaptic density	NA	[142]
Rho-kinase ROCK	Fasudil	6-9 m; M; rTg4510	Orally; 12 mg/kg	4w	NA	↓ levels of t-tau, p-Ser396/Ser404-Tau, caspase-cleaved tau, and oligomeric tau in sarkosyl-insoluble tau	NA	[143]

*↑* increased, *↓* decreased, *NA* not available or unknown, *ND* Not defined, *MWM* Morris water maze, *RAWM* Radial arm water maze, *NS* Non-significant, *FCT* Fear Conditioning Test, *NORT* Novel object recognition test, *b.w.* body weight, *OFT* Open Field Test.

^a^Data curated from PubMed, Scopus and Google Scholar as of July 1, 2022.

^b^Daily unless stated otherwise.

The 17 shortlisted studies targeted GSK3β (via SAR502250 [132]; tideglusib [130]; lithium chloride [133, 179, 180]; AR-A014418 [133]; MMBO [135]); CDK-5 (via ®-roscovitine [136]), p38MAPK (via MW181; SB239063 [137]); c-Abl and Src (via nilotinib [138]; bosutinib [138]), Fyn (AZD0530 [181]), DYRK1A (via Dryk1-inh [140]; DYR219 [141]; SM07883 [139]), MARK4 (dl-NBP [182]), SYK (BAY61 [142]) and Rho-kinase (Fasudil) [143] inhibition, which resulted in cumulative reduction of tau hyperphosphorylation and alleviation of the AD cognitive/behavioural phenotype.

The studies were performed utilizing transgenic mouse models: 3xTg-AD, JNPL3 (P301L), PS19 (P301S), APP^sw^-Tau^vlw^, PrP T44 Tau, and hTau and rTG4510. The commonly used triple mutant 3xTg-AD mouse model contains two mutations associated with familial AD, APP (KM670/671NL; Swedish), and PSEN1 (M146V), and one with fronto-temporal dementia (FTD), MAPT (P301L) [183]. The double-transgenic APP^sw^-Tau^vlw^ mice over-express human mutant APP (KM670/671NL; Swedish) and triple mutant human MAPT (G272V, P301L, R406W) mimicking several features of the AD phenotype to a remarkable extent [184]. However, these combinations of mutations do not actually exist in AD. The JNPL3(P301L) mouse model expresses mutant human MAPT (4 R/0 N; P301L), where the hemizygotes express human tau at levels comparable to endogenous murine tau and homozygotes at approximately twice the endogenous levels [185]. The PS19 mouse has P301S tau (4 R/1 N) mutant overexpressed fivefold higher than that of endogenous tau. It develops tangles in the neocortex, amygdala, hippocampus, brain stem and spinal cord at 6 months, with progressive accumulation thereafter [186]. The rTg4510 mice express mutant human *MAPT* (4 R/0 N; P301L) downstream of a tetracycline operon–responsive element (TRE), to an activator line expressing a tetracycline-controlled transactivator under control of the CaMKIIα promoter [187]. The expression is largely restricted to the forebrain and can be inactivated by administration of the tetracycline analogue doxycycline [188]. These mutations are not present in AD, though.

To address these limitations, models such as PrP T44 Tau, and hTau mice were used; PrP T44 Tau mice express the shortest human tau isoform (T44, also known as fetal tau) [189] and hTau mice express all six classic human tau isoforms with endogenous murine tau knocked out [190]. Among these, the 3xTg AD model shows tau pathology in the hippocampus, particularly pyramidal neurons, where JNPL3(P301L) homozygous and heterozygous mice and P301S mice develop neuronal inclusions reminiscent of tangles and Pick-bodies by 4.5 and 6, and 6 months respectively [185, 186]. APP^sw^-Tau^vlw^, PrP T44 Tau, hTau, and rTG4510 mice line develop pathology in various brain areas: APP^sw^-Tau^vlw^ model manifests neuritic plaques [184], PrPT44 model develops tau-rich filamentous inclusions in cortical and brainstem neurons at 6 months [189], and hTau model develop aggregates and PHF detectable at 9 months [190] and rTG4510 accumulate an early burden of tau pathology in the form of argyrophilic inclusions, with tangles being observed in the cortex by 4 months and in the hippocampus by 5.5 months [187, 188]. Tau inclusions are characterized by Gallyas silver staining, Bielschowsky silver staining, Thioflavin S, or Congo red and presence of sarkosyl-insoluble tau. Neurofibrillary lesions correspond to motor and/or cognitive decline, associated neurodegeneration and neuroinflammation [191].

The efficacy of PKI-treatment was examined in the above studies at a behavioural, biochemical, and histological/immunochemical level, with methods tailored to the impairment observed in individual models. With respect to the behavioural aspect, the cognitive functions such as learning and memory were assessed using Morris water maze (MWM), radial arm water maze (RAWM), novel object recognition test (NORT), open field test (OFT), fear conditioning test (FCT), T-/Y-maze, or Barnes maze (BM). Motor functions and deficits were assessed using wire-hang test, whereas to assess anxiety and depression-like behaviour, forced-swim or tail suspension tests were used. Improvements in cognitive functions of learning and memory were assessed by improved escape latency in the MWM for tideglusib [130], Dyrk1-inh [140], and partially dl-NBP with increased freezing time in FCT [182], improved performance on NORT for SAR502250 [132] and MMBO [135], improvement in hippocampal dependent spatial working memory deficits in Y-maze test for MW181 and SB239063 [137]. Motor functions demonstrated improvement in clasping phenotype in the tail suspension test for LiCl [133, 179, 180]. Anxiety and depression-like behaviour showed effective improvement in the wire suspension test for SM07883 [139]. In the remaining studies, the animals were not subjected to behavioural testing.

In biochemical, histological and immunochemical analysis, almost all inhibitors demonstrated dose-dependent or independent decrease in hyperphosphorylation of tau at sites that were specific for the inhibited kinases, which is indicative of the pharmacodynamic action of these inhibitors directly on tau. For LiCl [180] and SB239063 [137] biochemical analysis were not performed, thus their pharmacodynamic effect on tau hyperphosphorylation remains unknown. Altogether, up to 13 phospho-sites were analysed in these preclinical efficacy studies (*Ser199, Thr181, Ser262, Ser396, Ser202, Thr205, Thr212, Ser214, Thr231, Ser235, Ser396, Ser404 and Ser422*). It is worth noticing that although tau has ~85 putative phospho-sites and a large portion of them (>40 sites) are found to be hyperphosphorylated in AD, of which each kinase targets a handful of sites that it can phosphorylate, the choice of analysed phosphor-sites (as low as one or two in some studies [132, 141]) limits the interpretability of these results. In addition, the lack of consideration of the PK in question and their respective p-sites was also apparent in some studies.

As tau hyperphosphorylation is hypothesised to lead to aggregation, confirming the reduction of detergent-insoluble tau is of utmost importance; it is imperative that studies show a clear impact of kinase inhibition on the reduction of oligomeric and filamentous tau, as mere tau hyperphosphorylation could be a physiologically protective phenomenon. In this context, LiCl [180], MW181 and SB239063 [137], SM07883 [139], and Fasudil [143] demonstrate significant reduction in levels of sarcosyl-insoluble tau. Meanwhile, with Tideglusib [130], the levels in the selected age groups were pointed out to be too low to be reliably quantified [192, 193]. BAY-61 [140, 142] and AZD0530 [181] were also demonstrated to reduce detergent and tris-buffered saline (TBS) insoluble fractions. In the remaining studies, although the data from immunoblots was consistent with immunohistochemical staining, demonstrating efficient reduction in tau hyperphosphorylation [137, 180], yet the quantification of insoluble fractions was not performed. In the process of evaluating the efficacy of the compounds in selected preclinical studies, a critical limitation remained their inability to assess the effect of the studied compounds on the tangle load (i.e., mature tau pathology) which represents a likely prerequisite for successful therapy of AD and related tauopathies. It may be overly ambitious to require a treatment to remove existing neurofibrillary pathology, but compounds should be able to reduce the accumulation of new neurofibrillary lesions.

From a methodological point of view, many studies are either flawed, or do not report on key aspects such as methods to counteract bias. Sample size calculations are missing across all studies. Randomisation is reported for a few studies [137, 139, 180–182], however, the method of randomisation is not reported. Blinding of the experimenters to treatment groups, a key step in preventing bias, was limited to a few studies [137, 138, 140, 143, 180]. Sex proportionality is maintained at large [130, 133, 136–139, 142, 179, 180, 182].

To conclude, future studies should most importantly assess the effect of putative therapies based on their effect on neurofibrillary pathology load as a primary outcome measure, and incorporate important design elements [194] such as effect size estimates and sample size calculation, the method of randomisation and allocation concealment, blinding, sex proportionality, exclusion and inclusion criteria (weight, health of animals…), in order to increase their rigour, transparency, and probability of succeeding in clinical development.

## Clinical development of protein kinase inhibitors and phosphatase modulators for Alzheimer’s disease

We have evaluated the available data from clinical trials for kinase inhibitors, with special emphasis on efficacy and safety of PK-targeted therapeutics for AD, and the druggability of targeted PKs. To this end, we conducted a comprehensive search through ClinicalTrials.gov, the Cochrane Central Register of Controlled Trials (CENTRAL), and the EU clinical trial register; and queried to identify Phase I/II/III/IV clinical trials testing PKIs against conditions mentioned as mild to moderate Alzheimer’s disease and/or Alzheimer’s disease in addition to mild cognitive impairment (MCI). Within our selected time frame, as of 1st July 2022, 26 clinical trials were completed, suspended, terminated, or withdrawn; these trials targeted only a handful of kinases. Only 10 trials tested direct effects on tau hyperphosphorylation and had changes in Tau-PET or plasma/CSF biomarker assessment of tau as their primary or secondary measures. These trials tested lithium, tideglusib, saracatinib and nilotinib. Notably, other potentially interesting kinase inhibitors, such as neflamapimod and valproate, were never evaluated in a study suitable to assess their impact on tau hyperphosphorylation in AD. All identified trials were interventional, randomised, placebo controlled, with parallel arm design, and were double-, triple, or quadruple-blind Table 3.

**Table 3:** Completed clinical trials exploring protein kinase inhibitors as tau-targeting disease modifying therapies for Alzheimer disease^a^.

Kinase	Inhibitor	Sponsor	Study identifier	Start date- Actual; estimated end date	Phase	Treatment duration	Population; age group	Conditions	Outcome measures	Observations	Ref.
GSK3β	NP031112 (Tideglusib/ NP12)	Noscira SA	NCT00948259	December, 2008 - November, 2009	1,2a	20 w (4 & 6 w)	30; 60–85 y	Mild-to-Moderate AD	*Primary* Safety assessment: (*AEs; ECG; vital signs; haematology; serum biochemistry; urinalysis*)	Safe and well tolerated with mild to moderate LFT abnormalities	[195]
									*Secondary* Clinical efficacy: Cognitive (*ADAS-Cog +; MMSE;* *WFT*), depressive (*GDS*) & psychometric (*GCA*) assessment	No improvement	
		Noscira SA	NCT01350362	April, 2011- October, 2012	2b	26 w	306; 50–85 y	Mild-to-Moderate AD	*Primary* Clinical efficacy: Cognitive (*ADAS-Cog*_*15*_) assessment	No improvement	[196]
									*Secondary* Safety and tolerability assessment: (AEs); Clinical efficacy: Cognitive (*ADAS-cog*_*11*_*, MMSE, ADCS-ADL; WFT*), behavioural (*NPI; GDS*), CSF biomarkers (t-tau; p-tau; Aβ_1-*42*_; BACE1), cerebral atrophy (*MRI*), urinary control (*QUI*), activities of daily living & quality of life (*CGI; EQ-5D; RUD Lite*), assessment	Safe and tolerable with SAE of ALT increase No improvement with an inconclusive trend in levels of pT181-tau	
	Lithium & Divalproex	National Institute of Neurological Disorders &Stroke (NINDS)	NCT00088387	July, 2004 - March, 2005	2	6 w	35; 40–90 y	AD	*Primary* Safety and tolerability assessment: (*LISER*; *AEs*)	NA	NA
									*Secondary* Clinical efficacy: Cognition (*MMSE*), & functional disabilities (*BADLS*) assessment	NA	
	Lithium Sulphate	Astra Zeneca (Sweden)	ISRCTN72046462	November, 2004 - July, 2005	2	10 w	71; 50–85 y	Mild AD	*Primary* Pharmacological assessment: GSK3 activity in lymphocytes; Clinical efficacy: CSF biomarkers (*pTau181; pTau321*) assessment	No effect of GSK3 activity No improvement	[198]
									*Secondary* Clinical efficacy: CSF biomarkers (*t-tau;* Aβ_1-*42*_), cognitive (ADAS-Cog; MMSE), & behavioural (NPI) assessment	No improvement	
	Lithium Carbonate	KCL	GO300913	December, 2004 - NA	2	12 m	22; <60 y	Mild-to-Moderate AD	*Primary* Safety assessment: Safety (adapted LISER)	Safe and well tolerated	[199]
									*Secondary* Clinical efficacy: Cognitive (*MMSE; ADAS-Cog*), functional (*BADLS*), behavioural (*NPI*), & global deterioration (*GDS*) assessment	Results inconclusive	
		University of Sao Paulo	NCT01055392	March, 2007 - March, 2009	2	24 m	80; 60–80 y	MCI; AD	*Primary* Clinical efficacy: Cognitive (*CDR-SoB; ADAS-Cog*), memory (*CERAD; delayed recall test; SLN; TMT*), & CSF biomarkers (t-Tau, p-Tau & Aβ_1-42_) assessment	No improvement	[200]
									*Secondary* Clinical efficacy: Conversion from aMCI to AD Safety & tolerability assessment: (AEs)	Results inconclusive Safe and well tolerated	
		New York State Psychiatric Institute	NCT02129348	June, 2014 - January, 2020	2	12 w	77; Child, Adult, Older Adult	AD; Psychosis; Agitation.	*Primary* Clinical efficacy: Psychiatric (*NPI -aggression domain score*) assessment	No improvement	[202]
									*Secondary* Clinical efficacy: Behavioural (*NPI core score; CGI; YMRS; TESS; SAS; BADL; ZCBI*) assessment	No improvement	
Fyn & BCR/Abl	AZD0530 (Saracatinib)	Stephen M. Strittmatter, Yale University	NCT01864655	July. 2013 – November, 2014	1b	4 w	24; 50–90 y	AD	*Primary* Safety & tolerability assessment: (*AEs);* Pharmacokinetics: CNS availability after oral dosing	Safe and well tolerated Sufficient	[204]
									*Secondary* Clinical efficacy: Cognitive (*ADAS-Cog; MMSE; ADCS-ADL; CDR-SOB*), behavioural (*NPI*), brain glucose metabolism (*FDG-PET*) assessment	No improvement	
		Yale University	NCT02167256	December, 14 - February, 2018	2a	52 w	159; 55–85 y	Mild AD	*Primary* Safety & tolerability assessment: (*AEs*), & brain glucose metabolism (*18F-FDG PET*) assessment	Safe and well tolerated No effect on 18F-FDG PET	[203]
									*Secondary* Clinical efficacy: Cognitive (*ADAS-Cog11; MMSE; ADCS-ADL; CDR- SoB*), behavioural (*NPI*), percent change in brain volume before & after treatment (*MRI*); CSF biomarker (*t-tau, p-tau, & Aβ*_*1-42*_) assessment	No improvement	
c-Abl	Nilotinib	Georgetown University	NCT02947893	January, 2017 - February, 2020	2	26 w	42; 50–85 y	AD	*Primary* Safety & tolerability assessment: (*AEs;* SAEs), Pharmacokinetics: CNS availability after oral dosing	Safe & well tolerated Sufficient	[205]
									*Secondary* Clinical efficacy: CSF biomarker (*Aβ*_*1-42*_*, Aβ*_*40*_, *p-tau181, t-tau*), CNS amyloid burden (*Florbetaben PET*), CSF, hippocampal volume (*MRI*), cognitive (*MMSE; ADAS-Cog; ADCS- ADL; CDR-SoB*), & behavioural (*NPI*) assessment	Results inconclusive	

*↑* increased, *↓* decreased, *NA* not available or unknown, *AEs* Adverse events, *SAEs* Severe adverse events, *LFT* Liver function tests, *WFT* Word fluency test, *ADAS-Cog+* Alzheimer’s Disease Assessment Scale - Cognitive subscale, *MMSE* Mini Mental State Examination, *GDS* Geriatric Depression Scale, *GCA* Global clinical assessment, *QUI* Questionnaire on urinary incontinence, *ADCS-ADL* Alzheimer’s Disease Cooperative Study Unit Activities of Daily Living, *BADLS* Bayer Activities of Daily Living Scale, *ALT* Alanine amino-transferase, *NPI* Neuropsychiatric Inventory, *CGI* Clinical Global Impression of Change, *EQ-5D* European Quality of life Instrument, *RUD Lite* Resource Utilization in Dementia-Lite, *LISER* Lithium Side Effects Rating Scale, *CDR-SoB* Clinical Dementia Rating scale - Sum of Boxes score, *TESS* Treatment Emergent Symptom Scale, *CERAD* Consortium to Establish a Registry for Alzheimer’s Disease, *SLN* Sequence of Letters and Numbers, *TMT* Trail Making Test, *YMRS* Young Mania Rating Scale, *SAS* Simpson-Angus Scale, *BADL* Basic activities of daily living, *ZCBI* Zarit Caregiver Burden Interview.

^a^Data curated from ClinicalTrials.gov, Cochrane Central Register of Controlled Trials (CENTRAL), EU clinical trial register, and ALZFORUM assessed on July 1, 2022.

GSK3β was targeted using *tideglusib, salts of lithium* and *valproate*. *Tideglusib* (NP-031112), an orally available drug with a thiadiazolidinone scaffold underwent two clinical trials. The pilot phase I/IIa trial (NCT00948259, *N* = 30, 4 & 6 weeks), was primarily designed to assess safety and tolerability. The drug was administered once daily (q.d.) at an escalating dose of 400, 600, 800, 1000 mg for 4, 4, 6, and 6 weeks respectively, in mild to moderate AD subjects. The overall safety findings in the study indicated that tideglusib can be administered for weeks/months if serum transaminase levels are closely monitored; due to the pronounced hepatotoxicity only 50% of the subjects continued the treatment though. Although there was no statistically significant evidence for efficacy, a difference of 4.7 points in the ADAS-cog+ and 1.7 points in the MMSE in favour of tideglusib was observed in subjects who could be escalated to 1000 mg [195]. A subsequent trial (NCT01350362, *N* = 306, 26w), called ARGO, tested daily doses of 500 mg and two different regimes of once a day (q.d.) and once every other day (q.o.d.) of 1000 mg; both doses demonstrated a reasonable safe profile, except 14–18% of subjects experienced diarrhoea, and 8.9–16% exhibited elevated transaminase levels. However, neither any significant long-term differences nor definite trends in any efficacy variable (ADAS-cog11, MMSE, Word Fluency test, ADCS-ADL, NPI, EQ-5D, and QUI) were observed, suggesting a lack of efficacy on the clinical front. In addition, CSF levels of Aβ_1-42_, total tau, pT181-tau and pS396-tau also remained unchanged, putting the expected pharmacological actions of tideglusib as a GSK-3β inhibitor in question. A significant reduction in BACE1 in the CSF of the treatment group was observed, but whether this had an effect on BACE1-mediated cleavage of amyloid precursor protein (APP) is uncertain. Overall, a non-significant trend was observed in the active treatment arm compared to placebo in the levels pT181-tau but the study was underpowered both to address the question of target-engagement of tideglusib, and to assess clinical efficacy [196]. Subsequently, tideglusib showed negative results in a trial in PSP [197].

*Lithium* is currently used as a mood stabiliser. A total of five completed phase II clinical trials in AD and one ongoing trial are on the record with *lithium salts*. Of these, only three have published their rather inconclusive results. Two short-term Phase II pilot clinical trials conducted to examine the acute effect of lithium on neuroprotective activity (ISRCTN2046462, *N* = 71, 10w) & (NCT00088387, *N* = 35, 6w) demonstrated no significant improvement in cognitive or neuropsychiatric measures (MMSE, ADAS-Cog score, NPI). The effect on GSK3β activity or concentration of blood or CSF derived biomarkers such as Aβ_1-42_, pT181-tau, pT231-tau and total tau were also not significant [198]. A subsequent open label trial (GO300913, *N* = 22, 1 y) with *lithium carbonate* (serum levels 0.3–0.8 mmol/l) produced no significant improvement on the MMSE in elderly individuals with mild to moderate AD. Lithium was deemed safe despite a high discontinuation rate, as the side effects were mild and reversible [199]. All these studies failed to find a positive effect of lithium on cognition, CSF p-tau and other AD-related blood or CSF biomarkers, conceivably because of small samples, short observation periods that were insufficient to detect any impact of neuroprotective agents, and the inclusion of subjects in more advanced stages of cognitive deterioration, i.e., mild, and moderate AD. In contrast, a later study (NCT01055392, *N* = 80, 2 y) sought to address the methodological aspects of trials on disease-modification in AD. It recruited patients with prodromal AD, i.e., amnestic mild cognitive impairment (aMCI) and administered a low dose (150 mg) of lithium. The statistical analysis performed by the authors doesn’t allow to assess whether treatment slowed progression over time; however, authors report a non-significant trend towards decrease of CSF p-tau (Fisher’s, *P* = 0.2) [200]. Another trial (NCT02129348, *N* = 77, 12 w) aiming to evaluate the efficacy and side-effects of low dose (150–600 mg) of lithium to treat agitation in AD subjects reported an excellent safety profile. GCI behaviour change on lithium (31.6%; *n* = 12) was observed to be similar to placebo (20.5% [*n* = 8]; X2(1) = 0.72; *P* = 0.40); however, there was moderate or marked improvement (CGI) in the lithium group (10/38 = 36.8%) compared to placebo (0/39 = 0%, Fisher’s exact test *p* < 0.001) [201, 202]. An actively recruiting Phase IV clinical trial LATTICE (NCT03185208, *N* = 80, 2 y) involving lithium carbonate as a possible treatment to prevent cognitive impairment in the elderly may further elucidate the effect of long-term lithium administration on CSF p-tau.

*Saracatinib* and *nilotinib* are small molecule inhibitors of Fyn and Bcr-Abl, respectively. In phase Ib and II clinical trials (NCT01864655, *N* = 24, 4 w & NCT02167256, *N* = 159, 52 w) *saracatinib* was found to be reasonably safe and well tolerated in participants with mild AD, but neither showed any statistically significant treatment differences for change in either CSF total tau or p-tau, nor impact on any other primary or secondary outcomes (cognition, brain glucose metabolism) over the course of treatment [203, 204]. *Nilotinib’s* phase II trial (NCT02947893, *N* = 42, 26 w) showed only an effect on CSF amyloid, CNS amyloid in the frontal cortex (but not temporal), and the level of the dopamine metabolite homovanillic acid (HVA) compared to the placebo group; no effects on tau were observed in the comparison between treatment arms. The major limitation of this study remains the lack of multiplicity adjustment, and other flaws in statistical analysis [205]. An active large-scale Phase III trial of nilotinib (NCT05143528, *N* = 1275, 5 y) is currently recruiting participants with early AD, who will be randomly assigned to receive 1 of 2 different doses (84 and 112 mg) of nilotinib or placebo for 72 weeks. A biomarker sub-study will investigate the effects of drug on the amyloid brain burden as well as tau and other markers of AD pathology.

*Sodium selenate* is the first drug targeting PP2A in clinical trials in AD and related disorders. A Phase IIa clinical trial compared sodium selenate (VEL015) to placebo or low-dose selenate in 40 subjects with mild–moderate AD over 24 weeks [206]. Sodium selenate was found to be generally safe, with minor side effects of headache, fatigue and nausea. No significant differences in cognitive measures between groups over the treatment period were observed, though. Additional exploratory diffusion-weighted MRI endpoints found less degeneration in the white matter of patients treated with sodium selenate than placebo [206]. Furthermore, a pooled analysis of all subjects showed that the patients who had higher selenium levels in their blood and cerebrospinal fluid (CSF) showed less cognitive decline than those with lower selenium levels [207]. The open-label extension study was not conclusive. A new phase II trial on sodium selenate in behavioural variant of FTD will use higher doses (15 mg taken three times daily) in a cohort of 120 patients [208, 209]. This trial is currently in recruitment and is expected to be completed by 2024. In summary, reduced phosphatase activity is likely responsible for a part of tau hyperphosphorylation. Due to the paucity of clinical evidence, it is too early to say whether enhancing phosphatase activity can yields an efficacious treatment.

It is important to note that published preclinical studies did not bring conclusive results on therapeutic effect on prevention of tangle formation or diminishing of tangle load by inhibiting tau kinases in transgenic animal models. Therefore, it is important to address this point in the clinical development by using suitable biomarker assessment as prescribed by the amyloid/tau/neurodegeneration (ATN) classification [210]. None of the above studies have a complete A/T/N classification for the whole patient cohort, which is particularly peculiar for e.g., the most recent study of nilotinib, which screens all patients for amyloid, yet does not appear to confirm tau pathology for inclusion. Recent results show that even mild AD dementia cohorts that are amyloid-positive can be tau-negative in ~30% of cases, hampering the efficacy readouts of tau-targeted compounds [211]. Currently, whether kinase inhibitors can efficiently halt the clinical progression of the disease via preventing the tangle formation cannot be conclusively stated. Most of the studies were not designed to answer questions of efficacy—the sample sizes were not sufficient to evaluate clinical endpoints with any degree of rigor. Studies on the various immunotherapeutic approaches, such as monoclonal antibodies, universally arrive at the conclusion that sample sizes of several hundred subjects are necessary to evaluate clinical efficacy of disease-modifying therapies for AD [212, 213]. Sample sizes required for biomarker assessments are usually smaller, yet many of the studies conducted on PKI were likely underpowered even in this aspect. The duration of observation in most of the discussed trials was also insufficient. A disease-modifying therapy, much unlike symptomatic treatments (e.g., acetylcholinesterase inhibitors), will not manifest its effects in a few months’ time. Even if a therapy stopped all neurodegeneration caused by tau pathology on day one of treatment, this would not become readily apparent until a concurrently randomised placebo control group declines. The reasons are manifold—a part of decline is driven by various common comorbidities such as vascular, TDP43 [214], or alpha-synuclein pathology [215]; the cognitive performance of patients varies on a day-to-day basis [216]; the clinical assessment tools are not perfect [217]; the margin of error of biomarker assessment methods is frequently close to the mean annual change on various disease markers such as hippocampal volume [218]; placebo groups often don’t decline right away. In fact, of the above, solely NCT01055392 with its 24-months had a duration usual for studies of potentially disease-modifying compounds [219]; the 36-moth duration chosen for the most recent nilotinib trial is also appropriate for an early AD population. As for populations selected for evaluation of these compounds, only a few confirmed the presence of tau pathology via CSF biomarkers or tau PET at enrolment, thus the question remains: what proportion of these subjects actually had tau pathology as their dominant neurodegenerative component? Finally, it must be mentioned that several of the studies had critical flaws in methodology, including incorrect statistical evaluation of the results, or claims of blinding where the study in essence was unblinded.

## Future drug development of tau hyperphosphorylation modulators

Further clinical studies are warranted to show whether this therapeutic approach is able to impact the clinical course of AD and non-AD tauopathies. To achieve this, numerous improvements in the design and methodology of these studies are quintessential. As for study populations, it should be obvious that to evaluate treatments that aim to impact tau hyperphosphorylation, one should select subjects whose neurodegeneration is primarily driven by tau pathology. Thus, inclusion criteria for trials should include a biomarker assessment that confirms this—CSF and/ tau and phospho-tau markers, tau PET, or blood tau markers. Markers of tau pathology and neurodegeneration should be evaluated longitudinally over a sufficient time frame. Markers that reflect current neurodegeneration intensity (e.g., CSF tau) can be expected to respond sooner than markers that reflect disease progression (e.g., tau PET); with the latter, expecting a small molecule to clear tau deposits present at baseline may be too ambitious, and expecting the compound to slow tangle accumulation may be more realistic.

When calculating sample sizes, especially for clinical endpoints one should consider a) that even if a compound halts the disease in its tracks, an improvement above baseline is not to be expected with disease-modifying compounds, b) even excellent drugs should not be expected to prevent all of the clinical decline in subjects. A slowing of the disease by 25% should be considered a success. Meanwhile, the design of many of the reviewed trials (sample size, observation period) indicates that investigators were expecting the subjects to not just slow or cease deterioration, but to improve above baseline.

Accordingly, trials should choose endpoints that are appropriate for the stage of development, i.e., at early development stages, a primary target engagement endpoint such as CSF phospho-tau is more appropriate than clinical endpoints. A robust effect on a tau marker in an early study should be valued higher than a seemingly massive (most likely coincidental) effect on a clinical endpoint in a tiny group. Consequently, the success criteria for phase 1 and smaller phase 2 studies should be primarily based on showing proof-of-mechanism, i.e., biomarker impact. Meanwhile, the efficacy in later-stage studies need to be established based on clinical endpoints, as no surrogate efficacy biomarkers are established yet.

As for lead selection for clinical development, compounds should be selected based on multiple criteria. Primarily, compounds should show a robust effect in proper models that actually recapitulate the development of neurofibrillary pathology. An aspect of pharmacokinetics that has often been neglected is that PKI intended for AD treatment need to have decent blood-brain-barrier penetration (see ref. [220])—which was not always the case for PKI repurposed from oncology, as seen with nilotinib.

As AD tau is hyperphosphorylated at more than 40 sites, it may be beneficial to study combinations of PKIs, or multi-target inhibitors, e.g., a combination of PDPKs and non-PDPKs [39]. In the ideal case, the combinations would include brain-specific kinases to limit systemic side effects. What should also be on the forefront of our thinking is the possibility of combining PKIs with modulators of phosphatase activity, constituting a two-pronged approach aimed at reducing hyperphosphorylation.

Finally, the studies should be adequately powered, and statistical rigor should be maintained; this especially applies to the assessment of cognition and function.

To conclude, the intimate connection tau hyperphosphorylation has to tau pathology genesis and progression is apparent in numerous preclinical studies. The translation to clinical practice has so far eluded grasp, with the entirety of the available evidence being insufficient to conclude unequivocally whether PKIs are efficacious in AD and tauopathies. It is definitely warranted to pursue this therapeutic concept further, but with adequate rigor, and employing the lessons gathered from the many failures in AD drug development.

### Supplementary information

Preclinical efficacy studies and clinical trials on PP2A activators
