# Supplementary material for: Does modulation of tau hyperphosphorylation represent a reasonable therapeutic strategy for Alzheimer’s disease? From preclinical studies to the clinical trials
Source: Mol Psychiatry. 2023 Jun 2;28(6):2197–214. doi: 10.1038/s41380-023-02113-z (PMC10611587; doi:10.1038/s41380-023-02113-z)
Supplement: Supplementary file 1 — Preclinical efficacy studies and clinical trials on PP2A activators [file 41380_2023_2113_MOESM1_ESM.docx]

**Supplementary tables**

***Table S1.*** *Shortlisted efficacy studies on selected PP2A activators.*

| **Activators of PP2A** | **E4** | **Age; sex; animal model** | **Administration; Dosage** | **Treatment** | **Results** | | | **Ref.** |
| --- | --- | --- | --- | --- | --- | --- | --- | --- |
|  |  |  |  |  | **Behavioural test** | **Biochemical analysis** | **Histopathology/ Immunochemistry** |  |
| **Glimepiride** | ATP-sensitive K+ channel | 3m; M; P301S mice | Orally; 1, 2, 4 mg/kg | 22d | ↓ anxiety-like behaviour in OFT and motor impairment in PRFT | ↑ PP2A protein level | ↓ neurodegeneration and histopathological changes  ↓ pSer396-tau | [^1^](#_ENREF_1) |
| **Copper/zinc chaperone (PBT2)** | ND | 11m; M,F; rTg4510 | Oral gavage; 30mg/kg | 6w | Improved memory in Y-maze | No change in t-tau  ↓ pSer396-tau  ↓ sarkosyl-insoluble-tau | ↓ NFTs in hippocampus and frontal cortex | [^2^](#_ENREF_2) |
| **Clioquinol (CQ)** | ND | 9m; M; hTau mice | Oral gavage; 30 mg/kg | 5w | NA | ↓ pSer396-, pThr205-, pSer214-, and pSer404-tau | ↓ pSer404-tau | [^3^](#_ENREF_3) |
| **Sodium selenate** | PP2A C | 15, 5m; TMHT mice | Orally; 1.2 mg/ml | 12w | Improvement in spatial memory and escape latency in MWM | NA | ↓ NFTs and tau-positive cells  ↓ pThr231-tau  ↓ t-tau | [^4^](#_ENREF_4) |
|  |  | 9m; M; THY-Tau22 mice | Orally; 12 μg/ml | 12w | Increased activity and exploratory behaviour in CA & OFT; Improved escape latency in MWM; Improved memory retention in PA | NA | ↓ pSer202/Thr205-tau | [^5^](#_ENREF_5) |
|  |  | 3m; M; 3×Tg-AD mice | Orally; 6 μg/mL | 10m | NA | NA | ↓ pS404-, pS396- and pT231-tau in the hippocampus | [^6^](#_ENREF_6) |
|  |  | 6-8m; NA; K3 (pR5, and pR5.Dom5) mice | Orally; 12 μg/mL | 3m, 4m, 8m, 9m | Improving motor performance in RRT | ↓ pThr231/Ser235-, pSer422-tau  ↓ Sarkosyl insoluble-tau | ↓ pThr231/Ser235-, pSer422-tau  ↓ inclusions | [^7^](#_ENREF_7) |
| **S-adenosylmethionine** | LCMT-1 | 6m; M, F; rTg4510 mice | Orally; 100 mg/kg | 21d | Rescued spatial memory in Y-maze | ↓ pSer202/Thr205-tau | ↓ pSer202/Thr205-tau | [^8^](#_ENREF_8) |
| **Metformin** | PP2A C | 2.5 m; M, F; PS19 inoculated with end-stage PS19 sarkosyl extract | Orally; 4 mg/ml | 4m | Improved escape latency in MWM | ↓ pSer202/Thr205, pSer262, pSer396, pThr231, and pSer422 ipsilateral Sarkosyl-insoluble Tau;  ↓ pSer202/Thr205 and pSer422 in Sarkosyl-insoluble Tau | ↓ tau seeding  ↓ pThr231 in the ipsilateral cortex  ↓ pS202/pT205-, pThr231-, pSer422-tau in the contralateral cortex | [^9^](#_ENREF_9) |
|  |  | 4w; P301S mice | Orally; 2 mg/ml | 4m | NA | No change in t-tau;  ↓ pSer262, pSer202/Thr205, pSer396/Ser404 and pThr181; | ↓ pSer202/Thr205-tau | [^10^](#_ENREF_10) |
| **Caffeine** | ND | 2m; M: THY-Tau22 mice | Orally; 0.3 g/l | 10m | Improved performance in MWM | ↓ pSer212/Thr214-, pSer396- and pSer422-tau;  ↓ proteolytic tau fragments | NA | [^11^](#_ENREF_11) |
| **Fingolimod** | S1P1 | 6-12m; M, F; 3xTg AD mice | Orally; 1mg/kg | 2 and 6m | Improves memory in NOL and escape latency in MWM | ↓ pSer202/Thr205-tau | NA | [^12^](#_ENREF_12) |

*Drugs/compounds that have been shown to alleviate aberrant tau phosphorylation in a PP2A-dependent manner. Note that most of these compounds, which reduce tau phosphorylation levels, act indirectly on PP2A. In many cases, the specificity of these compounds for PP2A has not been fully validated, and/or underlying PP2A regulatory mechanisms are unknown.*

**Abbreviations.**

*↑: increased; ↓, decreased; NA, not available or unknown.*

***MWM:*** *Morris water maze,* ***FCT:*** *Fear Conditioning Test,* ***OFT:*** *Open Field Test,* ***RRT****: Rota-Rod test,* ***PRFT****: Parallel rod floor test,* ***CA****: Cage activity,* ***PA****: Passive avoidance,* ***NOL****: Novel object location*

***Table S2.*** *Selected PP2A activators in clinical trials.*

| **Activators of PP2A** | **Sponsor** | **Study identifier** | **Start date- Actual; estimated end date** | **Phase** | **Treatment duration** | **Population; age group** | **Conditions** | **Outcome measures** | **Observations** | **Ref.** |
| --- | --- | --- | --- | --- | --- | --- | --- | --- | --- | --- |
| **Sodium Selenate (VEL015)** | Velacor Therapeutics | ACTRN12611001200976  (http://www.anzctr.org.au/) | December, 2011- June, 2013 | 2a | 24w | 40; M, F; ≥55 y; | Mild-to-Moderate AD | ***Primary***  Safety assessment: (*AEs; vital signs; physical examination, neurological examination, laboratory evaluations (haematology, biochemistry, and urine analyses), and ECG* | Safe and well tolerated with mild to moderate LFT abnormalities | [^13^](#_ENREF_13)^,^ [^14^](#_ENREF_14) |
|  |  |  |  |  |  |  |  | ***Secondary***  Clinical efficacy:  Cognitive (*ADAS-Cog; MMSE; COWAT; CFT; OCL, DET, IDN*),  CSF biomarker (*p-tau, t-tau, and Aß_1-42_*), neuroimaging metrics (*structural MRI, diffused-weighted MRI, FDG-PET*) | No improvement |  |

*Compound tested in Mild-to-Moderate AD patients with a clear mechanism of action of targeting PP2A activation.*

**Abbreviations.**

*↑: increased; ↓, decreased; NA, not available or unknown.*

***AEs****: Adverse events,* ***ECG****:* *electrocardiogram,* ***ADAS-Cog****: Alzheimer's Disease Assessment Scale - Cognitive subscale,* ***MMSE****: Mini Mental State Examination,* ***COWAT****: Controlled Oral Word Association Test,* ***CFT****:* *Category Fluency Test,* ***OCL****:* *one-card learning memory task,* ***DET****: detection reaction time task,* ***IDN****: identification reaction time task,* ***MRI****: Magnetic resonance imaging,* ***FDG-PET****: F-fluorodeoxyglucose-* *positron emission tomography.*

**Bibliography**

1. Zaki MO, El-Desouky S, Elsherbiny DA, Salama M, Azab SS. Glimepiride mitigates tauopathy and neuroinflammation in P301S transgenic mice: role of AKT/GSK3beta signaling. *Inflammopharmacology* 2022; **30**(5)**:** 1871-1890.

2. Sedjahtera A, Gunawan L, Bray L, Hung LW, Parsons J, Okamura N *et al.* Targeting metals rescues the phenotype in an animal model of tauopathy. *Metallomics* 2018; **10**(9)**:** 1339-1347.

3. Xiong Y, Jing XP, Zhou XW, Wang XL, Yang Y, Sun XY *et al.* Zinc induces protein phosphatase 2A inactivation and tau hyperphosphorylation through Src dependent PP2A (tyrosine 307) phosphorylation. *Neurobiol Aging* 2013; **34**(3)**:** 745-756.

4. Corcoran NM, Martin D, Hutter-Paier B, Windisch M, Nguyen T, Nheu L *et al.* Sodium selenate specifically activates PP2A phosphatase, dephosphorylates tau and reverses memory deficits in an Alzheimer's disease model. *J Clin Neurosci* 2010; **17**(8)**:** 1025-1033.

5. Ahmed T, Van der Jeugd A, Caillierez R, Buee L, Blum D, D'Hooge R *et al.* Chronic Sodium Selenate Treatment Restores Deficits in Cognition and Synaptic Plasticity in a Murine Model of Tauopathy. *Front Mol Neurosci* 2020; **13:** 570223.

6. Jin N, Zhu H, Liang X, Huang W, Xie Q, Xiao P *et al.* Sodium selenate activated Wnt/beta-catenin signaling and repressed amyloid-beta formation in a triple transgenic mouse model of Alzheimer's disease. *Exp Neurol* 2017; **297:** 36-49.

7. van Eersel J, Ke YD, Liu X, Delerue F, Kril JJ, Gotz J *et al.* Sodium selenate mitigates tau pathology, neurodegeneration, and functional deficits in Alzheimer's disease models. *Proc Natl Acad Sci U S A* 2010; **107**(31)**:** 13888-13893.

8. Beauchamp LC, Liu XM, Sedjahtera A, Bogeski M, Vella LJ, Bush AI *et al.* S-Adenosylmethionine Rescues Cognitive Deficits in the rTg4510 Animal Model by Stabilizing Protein Phosphatase 2A and Reducing Phosphorylated Tau. *J Alzheimers Dis* 2020; **77**(4)**:** 1705-1715.

9. Zhao S, Fan Z, Zhang X, Li Z, Shen T, Li K *et al.* Metformin Attenuates Tau Pathology in Tau-Seeded PS19 Mice. *Neurotherapeutics* 2022.

10. Barini E, Antico O, Zhao Y, Asta F, Tucci V, Catelani T *et al.* Metformin promotes tau aggregation and exacerbates abnormal behavior in a mouse model of tauopathy. *Mol Neurodegener* 2016; **11:** 16.

11. Laurent C, Eddarkaoui S, Derisbourg M, Leboucher A, Demeyer D, Carrier S *et al.* Beneficial effects of caffeine in a transgenic model of Alzheimer's disease-like tau pathology. *Neurobiol Aging* 2014; **35**(9)**:** 2079-2090.

12. Fagan SG, Bechet S, Dev KK. Fingolimod Rescues Memory and Improves Pathological Hallmarks in the 3xTg-AD Model of Alzheimer's Disease. *Mol Neurobiol* 2022; **59**(3)**:** 1882-1895.

13. Malpas CB, Vivash L, Genc S, Saling MM, Desmond P, Steward C *et al.* A Phase IIa Randomized Control Trial of VEL015 (Sodium Selenate) in Mild-Moderate Alzheimer's Disease. *J Alzheimers Dis* 2016; **54**(1)**:** 223-232.

14. Cardoso BR, Roberts BR, Malpas CB, Vivash L, Genc S, Saling MM *et al.* Supranutritional Sodium Selenate Supplementation Delivers Selenium to the Central Nervous System: Results from a Randomized Controlled Pilot Trial in Alzheimer's Disease. *Neurotherapeutics* 2019; **16**(1)**:** 192-202.
